# The General Practitioner

**Published:** 1920-10-30

**Authors:** 


### THE GENERAL PRACTITIONER.

In the pages of a journal devoted to the welfare of the hospital world and all the people's associations with a now time-honoured profession, there must always be a tendency to view with a somewhat conservative affection the achievements of the past, There is, of course, the historical as well as the moral interest to be derived from any medical retrospect, and we have always taken pleasure, at the expense of criticism by the ultra-modern, in attempting to extract those teachings from the past which the present is so ready to forget. At no time has the need for such reflection been so obvious as today. The almost chaotic condition into which the political stability of this country threatens to be plunged, the intricate causes of disaffection so glibly yet so inadequately described as "industrial unrest," call for the most deliberate review of the methods and thoughts upon which the past years of national prosperity were based.

If we turn to our own particular sphere of interest there are at the moment half a dozen keenly debated medical and institutional problems, the effect of which might be just as inadequately classed as a professional unrest. But here, on the other hand, there are abundant signs that needed thought and well-considered deliberation are actually taking place. Careful and collective institutional reorganisation, typified by the excellent work of Sir Napier Burnett, is progressing at a satisfactory, if not ultra-rapid, rate. The questions of Governmental or rate aid for the hospitals and of the extent to which the medical profession's work among the general public shall be directly and indirectly controlled by the State, are receiving a reasonably representative study from the various parties concerned. In these matters the average medical practitioner is perforce relying on his existing medical societies and associations. In the majority of cases their activities are for the moment well-known to him, and particularly he should appreciate the formation of the new Federation, which is bravely attempting to bring to the surface really representative medical opinion.

But the task which this body has before it now and indeed will ever have, might be materially lightened if the average medical man were in normal, as well as abnormal, times brought into closer association with the variety of official bodies which supposedly reflect his political and other opinions. There seems little doubt that for some time past, to not a few men, who are good and experienced practitioners, medical societies and associations have been steadily losing their interest. These meetings must be made more attractive to the average doctor. It is not too much to say that by attending many of the more recent meetings, often at the cost of valuable time, or when they could be taking well-earned recreation, general practitioners learn little that is of any practical value in the task of actual caring for their patients in civil life. Too often they listen to superficially attractive "preliminary reports" of researches emanating from workers whose experience, though great in their own sphere, is singularly limited where the first-hand care of the patient is concerned. The final appeal to laboratory work may be eminently satisfactory to those who specialise in that branch of medicine, but is a little lost upon those who have "borne the burden and heat of the day," and whose keenest desire is to know what is immediately useful and what is not. We see deplored by a man well qualified to judge, the fact that the older and established clinical methods are rarely pictured in their relation to disease as we see it to-day. After all, the appeal should be to the practitioner, and, in the outcome, to the patient.

